# Supplementary material for: Understanding the effects of real-time head position feedback on postural sway in terms of changes in underlying deterministic and stochastic dynamical processes
Source: PLoS One. 2025 Oct 30;20(10):e0334067. doi: 10.1371/journal.pone.0334067 (PMC12574899; doi:10.1371/journal.pone.0334067)
Supplement: Attachment — Submitted filename: PONE-D-24-33781 _ R.pdf [file pone.0334067.s001.pdf]

Dear Authors,

First, I would like to commend you on a well-executed study that presents a clear and rigorous experimental study on body sway across different sensory information conditions (eyes open, closed, and closed with vibrotactile feedback). The manuscript is both pedagogical and engaging, contributing valuable insights to the field. Your research question and hypotheses are well-articulated, and the study is methodologically robust, with findings that have been carefully presented and discussed.

I recommend accepting the manuscript with minor revisions. I particularly appreciate the adherence to a transparent presentation of results, regardless of impact level, aligning with the values of robust experimental research. Additionally, this study raises new avenues for research by addressing questions, such as the increased noise estimated by the Q metric in the model's diffusion term, which could lead to future investigations.

Below, I provide several constructive comments aimed at further enhancing the clarity and depth of certain sections.

1. **Cognitive Task Justification in Introduction**: The cognitive task's inclusion is a crucial aspect of your experimental design. While the discussion delves into this, expanding its rationale in the introduction would strengthen the manuscript. Currently, the factor 'task' in the ANOVA is briefly justified, creating an imbalance as its importance becomes evident only later. For instance, while the connection between constraint sources, attractor strength, and noise reduction through information richness is well-argued, the specific constraint of the dual-task demands remains less clear. In the introduction, you state (1) "that attractor strength estimates depended upon changes in task (i.e., standing while performing a text scanning task vs. just looking straight ahead)" (lines 178–179); (2) "Kinsella-Shaw et al. (24) found that RQA-based estimates of noise increased when the level of room illumination was decreased" (lines 180–181); and (3) "estimates of attractor strength derived from measurements of center of pressure fluctuations during stable standing decreased when participants were given an additional 'dual' task of counting backwards by 3's from a random starting number" (lines 192–194). However, later, you state: "Following Haggerty et al. (49) we hypothesized that the stabilizing effects of head position biofeedback would not be diminished by having participants perform a secondary 'dual' task while they were using the system" (lines 206–208). Shouldn't the dual-task condition affect the dynamics of body sway fluctuations in the EC+BF condition?
2. **Visual Information in Controlled Environments**: Another essential point is recognizing that, while open versus closed eyes provide clearly different levels of sensory information in natural contexts, this difference is less relevant in a visually controlled environment like the one used here. Toward the end of your manuscript, you note that "participants looked at a distal target on the far wall of a largely uncluttered room." It would be beneficial to address this limitation when describing information sources at the start. Open eyes in this setting do not fully replicate natural, enriched perception, a point very well raised later in the discussion.

3. **Carry-Over Effects and Guidance Hypothesis**: As I see it, the carry-over effects discussion could benefit from incorporating the guidance hypothesis [1, 2], which has also been observed with vibrotactile feedback when vision is available [3]. The consistency of your results with this literature is noteworthy, especially given the within-subjects design and the lack of an experimental group that only received vibrotactile feedback without vision across all trials because this would be the most direct way to determine if carry-over effects might occur in my opinion.
4. **Request for a Methodology Diagram**: A diagram illustrating the steps in the methodology would serve as a helpful summary of the procedure (e.g., training phase, self-selected trough width phase, etc.) including the number of trials in each phase, its duration and the average duration of the whole study for each participant. Such a visual representation would enhance the pedagogical clarity and allow readers to follow the method more intuitively.
5. **Minor Typos and Wording Suggestions**:
    - Line 229: "placed inside previously drawn foot outlines drawn (...)"
    - Line 244: "One marker was positioned on the back of a head (...)"
    - Line 277: "amplitude of vibration in each tactor vary with in medial-lateral head position (...)"
    - Line 296: "if they we not closely (...)"
    - Line 333: "The average medial-lateral head position over the three trials was used establish a (...)"
    - Lines 382–383: "settle on a setting that they felt best aided their ability to stand as still as possible" could be clarified by mirroring the discussion wording: "participants were tasked with finding the trough width that best aided their ability to stand as still as possible."

Thank you for considering these suggestions, which I believe will enhance the rigor and clarity of your well-structured and grounded study.

References:

1. Smeeton NJ, Huys R, Jacobs DM (2013) When less is more: Reduced usefulness training for the learning of anticipation skill in tennis. PLoS ONE 8(11): e79811.
2. Salmoni AW, Schmidt RA, Walter CB (1984) Knowledge of results and motor learning: A review and critical reappraisal. Psychol Bull 95: 355–386.
3. Lobo L, Travieso D, Barrientos A, Jacobs DM (2014) Stepping on Obstacles with a Sensory Substitution Device on the Lower Leg: Practice without Vision Is More Beneficial than Practice with Vision. PLoS ONE 9(6): e98801.
