# Supplementary material for: Understanding the effects of real-time head position feedback on postural sway in terms of changes in underlying deterministic and stochastic dynamical processes
Source: PLoS One. 2025 Oct 30;20(10):e0334067. doi: 10.1371/journal.pone.0334067 (PMC12574899; doi:10.1371/journal.pone.0334067)
Supplement: Attachment — Submitted filename: Harrison et al_Response to Review_R1.docx [file pone.0334067.s003.docx]

# Introduction to our response to reviews

Dear Editor and Reviewers

We are immensely grateful for your considerable efforts in carefully reading and reviewing our paper. Your comments and suggestions were very useful and have motivated major changes that have significantly improved the manuscript.

We describe the work we have done in response to your suggestions in detail below.

Thank you for your efforts

With thanks

Steven Harrison

# Response to Editor Journal Requirements

**Editor Comments on Journal Requirements 1:**

When completing the data availability statement of the submission form, you indicated that you will make your data available on acceptance. We strongly recommend all authors decide on a data sharing plan before acceptance, as the process can be lengthy and hold up publication timelines. Please note that, though access restrictions are acceptable now, your entire data will need to be made freely accessible if your manuscript is accepted for publication. This policy applies to all data except where public deposition would breach compliance with the protocol approved by your research ethics board. If you are unable to adhere to our open data policy, please kindly revise your statement to explain your reasoning and we will seek the editor's input on an exemption. Please be assured that, once you have provided your new statement, the assessment of your exemption will not hold up the peer review process.

**Author Response:**

We are working on a method of data sharing.

**Editor Comments on Journal Requirements #2:**

Please include your full ethics statement in the ‘Methods’ section of your manuscript file. In your statement, please include the full name of the IRB or ethics committee who approved or waived your study, as well as whether or not you obtained informed written or verbal consent. If consent was waived for your study, please include this information in your statement as well.

**Author Response:**

We have modified the text of our ethics statement and in our method section so that the text matches and contains the required information.

**Editor Comments on Journal Requirements 3:**

We are unable to open your Supporting Information file [Fig1.eps, Fig2.eps, Fig3.eps, Fig4.eps, Fig5.eps, Fig6.eps and Fig7.eps]. Please kindly revise as necessary and re-upload.

**Author Response:**

We have reexported these files.

**Editor Comments on Journal Requirements 4:**

Please include captions for your Supporting Information files at the end of your manuscript, and update any in-text citations to match accordingly. Please see our Supporting Information guidelines for more information: http://journals.plos.org/plosone/s/supporting-information.

**Author Response:**

NA

# Responses to Review #1

## Reviewer #1: Comment 1

Data on head displacement was converted into a haptic display in the form of vibrators in a headband. Young adults stood, wearing the sensors and vibrators. When the vibrators were in used, the spatial magnitude of sway was reduced. The authors expend a great deal of effort in analyzing and interpreting this effect. What they do not do is to explain why spatial magnitude is a good (much less ideal) metric for sway. Mechanistic and clinical approaches to postural control traditionally have simply assumed that “more sway” = “less stability”, but that assumption is no longer tenable. The authors also computed some dynamical aspects of sway but they seem to have little independent interest in these measures. The authors interpret the existing literature on “postural displays” in terms of access to “improved information”, viz., “Real-time postural biofeedback systems are technologies that appear to enhance our ability to detect information about the movements of our body, and as a consequence improve our capacity to control the posture of our body.” An alternative interpretation is that such displays simply provide a different suprapostural task, and that sway is tuned to facilitate performance of such tasks. The phrasing is ambiguous, but in the context of their focus on sway magnitude it may be that the authors assume that people always attempt to minimize sway. In revising, it will be essential to be clear about the authors’ position on this issue, not least because of the very large literature demonstrating that people do not always attempt to minimize sway magnitude.

## Author Response:

Your points here are well taken. We agree with the essence of all of them. We agree with you on the importance of task constraints. We agree with you that the equation “more sway” = “less stability” does not constitute a ubiquitous organizing principle for the human postural control system, and that people do not always attempt to minimize sway. We also agree that there is compelling data in the literature showing that increasing postural sway can be a way of improving information detection in the context of some tasks. That said, we believe that this is not likely in our experimental task where the explicit instructions are to maintain as fixed an orientation to the environment as possible, with the feet in a prescribed location and the head (gaze) fixated at a prescribed target. We believe that one requirement for satisfying this intention is to minimize sway that would displace the head.

Given this context, we can now answer a question you had in your comment, namely, why our chosen measurements of body sway and our chosen measures of the dynamics body sway are interesting and relevant metrics. In our experiment, and in a subset of everyday tasks, the constraints of the task operate such that a person’s goal is to organize one’s posture so that minimal body movement is generated. Expanding this logic to our use of an Ornstein-Uhlenbeck model of the dynamics of postural fluctuations, the constraints of the task operate such that a person’s goal is to approximate the dynamics of a critically damped system as closely as possible, by returning to equilibrium with as few oscillations around it as possible. This would be the optimal postural dynamics for any scenario where greater sway magnitudes were at odds with task demands, e.g., standing with a cup of hot coffee filled to the brim or when trying to blend into the scenery.

We interpret your comment here as a suggestion that these broader theoretical issues be made clear to a reader of our paper. With this end in mind, we have added a new section in the discussion titled: “Generalizability of findings and modelling approach”. The purpose of this section is to summarize the logic of our specific task and the task dynamic modelling approach in a way that provides a (hopefully clear) conceptual framework for readers to consider how to generalize our findings.

## Reviewer #1: Comment 2

The focus on “data driven” interpretations of postural control is puzzling given the emphasis found in the work of one of the authors on intentional dynamics which, if I understand that work, includes the claim that behavior happens, in part but always, in relation to goals. The authors might want to take such ideas more seriously in interpreting the present study.

## Author Response:

We are not entirely clear on what you are referencing when you express your concern that our manuscript has a “focus on “data driven” interpretations of postural control”. It is not a phrase we used. We interpret your comment to mean that you believe that our paper could be improved with a clearer presentation of the theory that frames the motivation for our research and the interpretation of our research findings. We believe that our new discussion section on “Generalizability of findings and modelling approach” does some useful work in this regard. It appears that you would like us to much more explicitly characterize the task constraints at play in our research. To do this, we have modified the current study section of our paper. We added a statement about what the overarching intention is, that is maintaining a posture that minimizes head movement relative to an eye-height visual target whether vision is available or not. In the current context, satisfying the task demands is synonymous with satisfying the intention, so any actions/behaviors that are consistent with the instructions to participants are reasonably assumed to be in service of what is intended.

The modified text reads as follows:

“In the current study, we examined how real-time feedback of medial-lateral head position (fig 1) would affect both the magnitude and dynamics of body sway fluctuations for young healthy adults instructed to “stand as still as possible”. Participants tried to maintain a posture with minimal head movement either with their eyes closed or with their eyes open gazing at a target presented at a fixed distance at the height of their eyes. Thus, the overarching intention across both these conditions was to maintain a maximally stable head orientation to the environment.”

## Reviewer #1: Comment 3

The haptic stimulators were positioned to reflect body sway in the mediolateral axis. Why? Most sway is in the anterior-posterior direction.

## Author Response:

We now include a description of the rationale for this design choice in the revised manuscript. It appears in the current study section of the introduction, and reads as follows:

“We provided feedback of medial-lateral head position using tactors positioned above the ears on the left and right side of the head (Fig 1A). Our choice to examine this positioning, rather than an anterior-posterior tactor placement, was motivated in-part by a wish to avoid the complications of introducing potential effects of vibration sensitivity asymmetries into our experiment. Vibration sensitivity asymmetries would likely have existed had we positioned the tactors on the anterior and posterior surfaces of the body (Corniani & Saal, 2020; Weinstein, 1968). The potential to modify postural sway in the medial-lateral direction is of particular interest to us for the following reasons: Changes in medial-lateral sway control has been reported as more predictive of an elevated risk of falls in older adults and neurologically impaired populations (e.g., patients with multiple sclerosis) compared to changes in anterior-posterior sway control ([Lord et al., 1999](https://www.sciencedirect.com/science/article/pii/S0531556522001115#bb0105); [Maki et al., 1994](https://www.sciencedirect.com/science/article/pii/S0531556522001115#bb0120); [Melzer et al., 2004](https://www.sciencedirect.com/science/article/pii/S0531556522001115#bb0145); Sidaway et al. 2022; [Stel et al., 2003](https://www.sciencedirect.com/science/article/pii/S0531556522001115#bb0215)). The effective regulation of medial-lateral body sway is known to be important for supporting basic human functions such as transitioning between standing and walking (i.e., step initiation in bipedal gait requires controlled lateral weight shifting) (Herman et al., 1973; Mann et al., 1979) and precise aiming (Balasubramaniam & Turvey, 2000). Lastly, even healthy younger adults, studied here, whose risk of injurious falls is considered minimal have been observed to show increased medial-lateral sway variability when visual support is significantly diminished or excluded (Kollegger et al., 1992; Rugelj et al., 2014).”

## Reviewer #1: Comment 4

In revising, the authors should include some justification for their sample size. Increasingly, such content is mandated in scholarly journals. Ideally, that would include an a priori power calculation, but as it appears to be too late for that the authors might at least include data on a posteriori power.

## Author Response:

We agree with the reviewer that a priori power calculation has a valuable place in research. We also appreciate the point that in hindsight, such power calculations could have been a nice addition to our approach. At your request, we now additionally report posteriori power (i.e., 1 − *β*) in the reporting of each of our statistical analyses.

## Reviewer #1: Comment 5

What is the difference between biofeedback and feedback? Is there any scientific content to the former term? Could we not do as well simply refer to the haptic display as providing feedback?

## Author Response:

Our choice to use biofeedback rather than feedback was motivated by its in related papers as a term connoting the use of feedback to control certain bodily processes that normally happen involuntarily. Our use of the term was also selected as a means of differentiating the feedback studied here from the very many uses of the term feedback in the literature (i.e., performance feedback at the end of a trial). We have no problem designating what the tactors provide as “feedback”. One benefit of this change is that in clinical parlance the usage of the term biofeedback is reserved for when information is being provided about an underlying and often autonomic process in the hopes that doing so would allow the patient to learn to control it more effectively. To make this change we have added clarifying language (e.g., real-time feedback of medial-lateral head position) in order to minimize possible confusion of the reader regarding the meaning of the word feedback.

## Reviewer #1: Comment 6a

The stance that the authors assessed was distinctly unnatural. Participants were compelled to keep their feet together, despite the fact that no one does this voluntarily. In other words, the study is about body sway in a non-preferred, unfamiliar stance. In revising, it will be necessary to acknowledge this fact (and its implications for interpretation of the data) and to provide an explicit motivation for it.

## Author Response:

In our new discussion section on “Generalizability of findings and modelling approach” we discuss the motivation for our experimenter introduced constraints. As we discuss in this section, what we take to be relevant to the generalizability of our results is not how someone is standing, but the emergent task dynamics promoted by the context of constraints (including how they are standing).

To directly address your request to present the logic of our choice to constrain stance, we have included the following text in the current study section of the introduction:

“Additionally, participants were to maintain the maximally achievable stable head orientation while standing with a narrow base of support, feet together symmetrically relative to the mid-line of the force platform. This postural constraint was introduced to minimize the range over which sway could occur before destabilizing a participant’s posture, moving them away from the intended head orientation. Standing in a symmetrical, “feet together” stance is a common component of clinical balance testing. A narrowed stance, reducing the base of support’s dimensions in the frontal plane has been shown to elevate control demands of stabilizing posture under a variety of conditions, including in the absence of visual support. Given that the participants were all healthy, young adults with no histories of balance impairments, this level of challenge was imposed to increase the likelihood that providing additional information for control, i.e., real-time vibrotactile feedback, would yield measurable differences in sway dynamics and magnitudes.”

In the context of natural settings, many circumstances arise in everyday settings when environmental constraints on actors compel them to adopt a wide range of foot placements, including standing on one foot, in order to satisfy the intention of maintaining upright posture with a given orientation to the environment. People are often required to assemble a narrower than normally preferable base of support while there is the concurrent goal of establishing a particular line of sight in the environment. Traversing a series of supporting surfaces that provide potential points of observation, such as stepping stones, ladders, stairs, etc.,all can require foot placement that differs from what a person would adopt if the goal were to simply stand on a planar surface of support with dimensions greater than the distances between their lower limbs. Additionally, people will often choose to stand with their feet very close together when in a crowd or when the goal is to draw themselves up to their full height for any reason, including improving lines of sight. Any stance that exploits what the local layout of surfaces afford such that an intention is satisfied constitutes a “natural” postural strategy.

## Reviewer #1: Comment 6a

Participants stood with their eyes closed. Why was this condition included? What predictions did the authors make about sway with the eyes closed, and what predictions did they make about relations between this condition and sway with the eyes open?

## Author Response:

Although we discussed our expectations regarding this manipulation in the discussion section of our original manuscript, we omitted explicitly stating our predictions in the current study section. We have corrected this error. In the current study section we now cover the logic of why we included these conditions, and our original predictions. The added text is as follows:

“In our study, we included two standard baseline conditions to facilitate our interpretation of the effect of real-time postural feedback. Specifically we included a standing with eyes open control condition, and a standing with eyes closed control condition. We predicted that the availability of visual information (i.e., standing with eyes open) compared to the absence of such information (i.e., standing with eyes open) would lead to 1) decrease the magnitude of body sway fluctuations (Riley et al., 1997; Kinsella-Shaw et al., 2006; Harrison et al., 2020), and 2) affect the dynamics of body sway fluctuations by increasing attractor strength (*λ*) and/or decreasing noise (Q) (Kinsella-Shaw et al., 2006; Riley et al., 1997).”

## Reviewer #1: Comment 6c

Participants were told to “stand as still as possible”. In revising, it will be necessary to provide an explicit justification for this requirement.

## Author Response:

See above

## Reviewer #1: Comment 7

The authors assert that in some conditions participants stood with eyes open and participants “focused only on standing balance”. This statement seems dubious. Presumably, what the authors meant was that the instructions given to participants referred only to stance, as such. But, if I understand the manuscript, the authors collected no data relating to “what participants focused on” and, given that their eyes were open it seems exceedingly likely that they were “focused”, at least in part, on what they could see.

## Author Response:

We edited this statement to hopefully remove its dubiousness. The edit is as follows:

“These three conditions were performed as a dual task, in which participants completed the auditory 3-back error identification task at the same time as performing the standing balance task, and as a single task where participants performed the standing balance task without simultaneously performing the auditory 3-back error identification task.”

## Reviewer #1: Comment 8

Please note also that when the authors refer to “dual task” conditions what they actually seem to be referring to is the instructions given to the participants (e.g., “do this and do that”). Again, there seems to be an implicit assumption that there is only one task (minimizing sway magnitude) unless the experimenter imposes a second task. In revising, please address the large, diverse literature that demonstrates otherwise (e.g., Haddad) and, separately, the use of postural activity to generate information, that is, exploratory sway (e.g., Hajnal, Palatinus). Among other things, the work of those (and other) scholars raises questions about the concept of “dual tasking”. It may be that postural and suprapostural tasks are integrated, such that they are not perceived or controlled as distinct “tasks”.

## Author Response:

We have added citations of the relevant work by Haddad, Hajnal, and Palatinus. Thank you for the suggestions. We agree with you that our usage of the term “dual-tasking” in our manuscript brings some theoretical baggage with it (i.e., some implicit assumptions about what constitutes a task) that has been called into question by some researchers. Given that the theoretical heart of this issue appears to be related to the question of what constitutes a task, we address the question of what constitutes a task and its connection to interpreting the concept of dual tasking at the end of the discussion section.

## Reviewer #1: Comment 9

Attending to the stimulators might simply have been a suprapostural task. That is, participants may have altered their sway so as to better detect the activity of the stimulators, rather than using the stimulators to reduce sway (Riley et al., 1999). If participants had been instructed to “stand comfortably” and told simply to notice (for example) the haptic stimulators, the results likely would have been different. Given this, we can wonder whether the authors’ arguments (and analysis, and conclusions) bear any meaningful relation to 1) ordinary stance (we rarely try to stand as still as possible) or to widespread, natural variations in the suprapostural tasks that are known (reliably, routinely) to influence both the spatial and temporal dynamics of sway. In general, the authors should explain how their study relates to the widespread (and ecologically motivated) argument that sway is not controlled solely—or even primarily—for its own sake but rather, that the avoidance of falling is integrated with the use of bodily movement in support of performance on suprapostural tasks.

- Riley, M. A., Stoffregen, T. A., Grocki, M. J., & Turvey, M. T. (1999). Postural stabilization for the control of touching. Human Movement Science, 18, 795-817.

## Author Response:

Your points here are well taken and appear to be closely related to the point you made in your comments about dual tasking above. Specifically, both this comment, and the one you made about dual tasking appear to be a request for us to address of the question of “what constitutes a task” in our interpretation of our experiment. We address the question of what constitutes a task and its connection to interpreting the concept of feedback aided balance at the end of the discussion section. This new section is titled “What are we studying here?”

## Reviewer #1: Comment 10

The Method section should be revised to provide much greater clarity about the total number of trials, the sequence of trials, the ordering of trials, and the duration of trials.

## Author Response:

To address this deficit in the manuscript we have 1) edited the method section to improve its clarity, and 2) added a new figure (fig 2) to overview the components of the procedure of the experiment.

## Reviewer #1: Comment 11

The final section of the Introduction, “Current Study” does nothing to motivate most of the experimental design. For example, the use of both standing and seated trials, and the use of both eyes open and eyes closed trials.

## Author Response:

Your question here did a good job of reinforcing the point you just made, that our somewhat complicated method was not clearly explained. There is no manipulation of seated vs standing. We believe that the revisions we mentioned above (i.e., adding fig 2 and editing the methods for clarity) will address this. Lastly, we have extensively edited the current study section to better motivate the research.

# Responses to Review #2

## Reviewer #2: Comment 1

**Cognitive Task Justification in Introduction**: The cognitive task’s inclusion is a crucial aspect of your experimental design. While the discussion delves into this, expanding its rationale in the introduction would strengthen the manuscript. Currently, the factor ‘task’ in the ANOVA is briefly justified, creating an imbalance as its importance becomes evident only later. For instance, while the connection between constraint sources, attractor strength, and noise reduction through information richness is well-argued, the specific constraint of the dual-task demands remains less clear. In the introduction, you state (1) "that attractor strength estimates depended upon changes in task (i.e., standing while performing a text scanning task vs. just looking straight ahead)" (lines 178–179); (2) "Kinsella-Shaw et al. (24) found that RQA-based estimates of noise increased when the level of room illumination was decreased" (lines 180–181); and (3) "estimates of attractor strength derived from measurements of center of pressure fluctuations during stable standing decreased when participants were given an additional ‘dual’ task of counting backwards by 3’s from a random starting number" (lines 192–194). However, later, you state: "Following Haggerty et al. (49) we hypothesized that the stabilizing effects of head position biofeedback would not be diminished by having participants perform a secondary ‘dual task’ while they were using the system" (lines 206–208). Shouldn’t the dual-task condition affect the dynamics of body sway fluctuations in the EC+BF condition?

## Author Response:

Thank you for this comment. In writing our original introduction we had focused our efforts on setting up the dynamical analysis (since seeing how postural feedback affects the dynamics of standing balance was the main aim of this study). In making this choice we now see that we failed to provide effective motivation for our dual task manipulation. To rectify this, we have significantly edited our current study section. We now give a much more thorough introduction to the manipulation of dual-task and our hypotheses related to this manipulation.

## Reviewer #2: Comment 2

**Visual Information in Controlled Environments**: Another essential point is recognizing that, while open versus closed eyes provide clearly different levels of sensory information in natural contexts, this difference is less relevant in a visually controlled environment like the one used here. Toward the end of your manuscript, you note that "participants looked at a distal target on the far wall of a largely uncluttered room." It would be beneficial to address this limitation when describing information sources at the start. Open eyes in this setting do not fully replicate natural, enriched perception, a point very well raised later in the discussion.

## Author Response:

Thank you for this comment. As with your comment earlier about our treatment of dual tasking, you correctly point out that our original introduction omitted a clear setting up of our manipulation of information. To rectify this, we have significantly edited our current study section. We now give a much more thorough introduction to our manipulation of information (i.e., EC, EO, EC+F) and our hypotheses related to this manipulation. We have chosen to leave the discussion of the limitations of our vision manipulations for the discussion section so that the paper can honestly reflect our oversight in designing the study.

## Reviewer #2: Comment 3

**Carry-Over Effects and Guidance Hypothesis**: As I see it, the carry-over effects discussion could benefit from incorporating the guidance hypothesis [1, 2], which has also been observed with vibrotactile feedback when vision is available [3]. The consistency of your results with this literature is noteworthy, especially given the within-subjects design and the lack of an experimental group that only received vibrotactile feedback without vision across all trials because this would be the most direct way to determine if carry-over effects might occur in my opinion.

## Author Response:

Thank you for pointing out this connection, it is insightful. We have integrated this point into our discussion section, in the final paragraph of the revised manuscript.

## Reviewer #2: Comment 4

**Request for a Methodology Diagram**: A diagram illustrating the steps in the methodology would serve as a helpful summary of the procedure (e.g., training phase, self-selected trough width phase, etc.) including the number of trials in each phase, its duration and the average duration of the whole study for each participant. Such a visual representation would enhance the pedagogical clarity and allow readers to follow the method more intuitively.

## Author Response:

Thank you for this suggestion. Some of the comments the other reviewer also made it clear that we had not done a good job in making it easy for the reader to understand our methods. To address this we have done some editing of the methods section for clarity, and as you suggested, we have created a new figure (fig 2) that describes the design of the study.

## Reviewer #2: Comment 5

**Minor Typos and Wording Suggestions**:

- Line 229: "placed inside previously drawn foot outlines drawn (...)"
- Line 244: "One marker was positioned on the back of a head (...)"
- Line 277: "amplitude of vibration in each tactor vary with in medial-lateral head position (...)"
- Line 296: "if they we not closely (...)"
- Line 333: "The average medial-lateral head position over the three trials was used establish a (...)"
- Lines 382–383: "settle on a setting that they felt best aided their ability to stand as still as possible" could be clarified by mirroring the discussion wording: "participants were tasked with finding the trough width that best aided their ability to stand as still as possible."

## Author Response:

Thank you for identifying these typos. We have address them all.
