# Supplementary material for: Understanding the effects of real-time head position feedback on postural sway in terms of changes in underlying deterministic and stochastic dynamical processes
Source: PLoS One. 2025 Oct 30;20(10):e0334067. doi: 10.1371/journal.pone.0334067 (PMC12574899; doi:10.1371/journal.pone.0334067)
Supplement: Attachment — Submitted filename: Response to Reviewers_R2.docx [file pone.0334067.s004.docx]

# INSTRUCTIONS

Please include the following items when submitting your revised manuscript:

- A rebuttal letter that responds to each point raised by the academic editor and reviewer(s). You should upload this letter as a separate file labeled '**Response to Reviewers'**.
- A marked-up copy of your manuscript that highlights changes made to the original version. You should upload this as a separate file labeled '**Revised Manuscript with Track Changes**'.
- An unmarked version of your revised paper without tracked changes. You should upload this as a separate file labeled 'Manuscript'.

# Introduction to our response to reviews

Dear Editor

We are immensely grateful for your considerable efforts in carefully reading and reviewing our paper. Your comments and suggestions were very useful and have motivated multiple changes that have significantly improved the manuscript yet again.

We describe the work we have done in response to your suggestions in detail below.

Thank you for your efforts

With thanks

Steven Harrison

# Response to Editor Comments

**Editor Comment 1:**

In addressing the comments by the original reviewers, some parts became quite long. For instance, the discussion of the dual-tasking in the Introduction (lines 273-293) can be shortened so that your manuscript can maintain its focus. Likewise, the Discussion has expanded considerably (e.g., lines 987-1108), and I would recommend to make these new parts more concise. In addition, the discussion of your main finding that head position feedback increases noise is important but some speculations become rather long (e.g., thermal noise). Please shorten parts that are rather speculative or can become more concise in order to maintain a sharp focus on your main findings.

**Author Response:**

We have dramatically shortened the section on dual-tasking in the introduction. To reduce the length of the discussion in the area you highlighted (i.e., lines 987-1108 in the track changes version of the first revision) we have completely removed the section on “What are we studying here?”. We believe that this section is not necessary for the paper. We have looked at the section on “Generalizability of findings and modelling approach”. We did not find any opportunities for shortening this section that did not negatively affect our ability to clearly lay out the logic of how we believe our findings could be generalized. Regarding the paragraph on thermal noise, we again did not find any opportunities for shortening this section that did not make the logic we were presenting opaque. We are hesitant to remove this section since noise is one of our main dependent variables and the logic in this paragraph helps frame a clear interpretation of our findings.

**Editor Comment 2:**

Related to these, please review your reference list and assess whether it is necessary to cite 128 studies to back-up the claims and connect with the previous work. This list feels quite long at the moment. In addition, some references may need more details to comply with the standards of citing a book or a book chapter (e.g., #113 and #115). Reference #74 has also a typo (year is mentioned twice).

**Author Response:**

We have corrected the Weinstein reference (Previously #74).

We have corrected the Riccio reference (Previously #113).

We have corrected the Hajnal reference (Previously #115).

We have also made minor corrections to multiple other references

Lastly, we have carefully gone through the paper and examined our references. We have identified and removed all references that appear redundant. We have removed a total of 17 references.

**Editor Comment 3:**

In lines 158-160 it is mentioned that larger λ represent greater attractor strength. However, in line 674 it is mentioned that "more-negative values" are associated with greater attractor strength. These are inconsistent as "more negative values" are "smaller", not "larger". Please clarify.

**Author Response:**

We have corrected this confusing wording. It is the case that more-negative values are associated with greater attractor strength.

**Editor Comment 4:**

Line 162 mentions that larger Q values indicate a faster diffusion process. Is this true? As far as it seems to me, Q indicates diffusion but this diffusion can occur in many ways (e.g., fast small fluctuations or many larger/slower ones). Please clarify whether Q indeed captures directly the speed of the fluctuation.

**Author Response:**

In the previous work (Bonnet et al) that developed and applied this modelling approach, a larger Q value is interpreted as indicating a more rapidly diffusion process. We have added this citation to the end of this sentence so that the reader understands where this interpretation of Q comes from.

**Editor Comment 5:**

It would be helpful if you would add a few comments as to whether the vibrations above the ears could impact the vestibular system, which in turn can influence balance.

**Author Response:**

We do not expect the tactor vibrations to influence balance. Our logic for this conclusion is as follows: Stated simply, the action of the tactors does not impose forces on the head that result in either rotary or linear displacements, so do not provide stimulation for the vestibular apparatus. The semicircular canals, require rotational displacements of the whole head of .7 to 2.0 degrees per second, depending on which axis of rotation is involved (“roll, pitch, and yaw”). The otolith organs, the vestibular subsystem that detects linear head movements, requires whole-head straight line displacements of .8 to 2.13 cm per second, depending on whether the movement is side-to-side, front-to-back, or up-to-down. Head displacements in any direction that are less than the magnitudes listed above are not adequate to displace the fluids or gels in the vestibular apparatus and so are subthreshold for registration.

We have added this information and a relevant citation to the paper.

**Editor Comment 6:**

Please report whether there were any differences between EO and EC in the ANOVA presented in lines 623-627. This comparison is misisng, suggesting that there is no effect. However, as there is a specific hypothesis about the role of vision on body sway (lines 234-237), this test should become clear. In addition, I would recommend to keep statistical procedures for testing the hypotheses as these are outlined in the introduction. Related to this, it is unclear why the effects of informational support on __*SDap*__ should be examined with an ANOVA (lines 634-641) considering that there is no hypothesis about body sway in AP direction. If there is a hypothesis, please add this in the Introduction and justify it. Otherwise, it would be suffiecient to show the related data in a figure (which seems to be missing).

**Author Response:**

We have added information about the pairwise comparison between EO and EC in the ANOVA of *SD*_ML_ values presented in lines 623-627 (your line numbers referring to the track changes version of the first revision). In regards to the effect of SDap, we had stated the hypothesis that “We expected to observe these predicted effects in the medial-lateral sway direction only (i.e., only in the direction of sway in which feedback was given)”. We have edited the language here to more explicitly and effectively highlight our hypothesis of directional specificity.

**Editor Comment 7:**

There are some tests in lines 727-729 that appear to be non-parametric. Please justify the use of non-parametric tests in certain cases.

**Author Response:**

In our previous manuscript we justified our use of a non-parametric test with the following statement: ”Given the high percentage of trials in which no errors were made, we used Wilcoxon Signed-Ranks Tests to compare CTES scores across conditions.” We have expanded upon this statement to make the logic more explicit. We now state that: “In all three conditions more than half of all obtained CTES scores had a value of 0.0 %. This meant that the distributions of CTES scores clearly violated the assumption of normality. We consequently used the non-parametric Wilcoxon Signed-Ranks Test to compare CTES scores across conditions.”

**Editor Comment 8:**

Finally, related to comment 6, I suggest keeping the part in lines 730-779 shorter and more concise. One possibility would be to present the three main ANOVAs in a unified manner and convey a single message. For instance, that "the feedback condition generally reduced the SD, and led participants to drift to their fixed point with greater noise". This is just a quick idea and far from optimal, but my point is to present these exploratory analyses in a shorter and more accessible form.

**Author Response:**

To clarify, the analyses in this section are not exploratory analyses. In our introduction we state the hypothesis that “We predicted that reducing trough width would both decrease the magnitude of body sway fluctuations and increase the attractor strength in the dynamics of postural fluctuations.” Given this hypothesis, it is necessary for us to report both the main effect and post-hoc pairwise comparisons. We have added some clarifying sentences to remind the reader of these hypotheses.

**Editor Comment 9:**

Figure 5, 6, 7 seem to be missing from the manuscript. Please provide them in the next submission.

**Author Response:**

We are not sure how they were ommitted. We will pay special attention when uploading them this time.

**Minor Comment from Editor 1:**
Line 431, a preposition seems to be missing: "..to sway __to__ the left..."?

**Author Response:**

We have added in the missing preposition.

**Minor Comment from Editor 2:**
Line 524: "Guassian" should be "Gaussian"?

**Author Response:**

This typo has been corrected.

**Minor Comment from Editor 3:**
Line 612: it would help if you would remind the reader about the levels of "information" and of "task".

**Author Response:**

We have inserted a reminder about the levels of task and information.

**Minor Comment from Editor 4:**
Line 801: this effect is mentioned in the previous line, I am not sure if it is necessary to be repeated.

**Author Response:**

We believe that these refer to different effects. The paragraph you reference on Line 801 summarizes the main effect of vision. The previous lines summarize the vision x biofeedback interaction effects.

**Minor Comment from Editor 5:**
Lines 859-861: this statement requires a citation. Please consider whether you can use any already existing citations to back this up, in light of an earlier comment related to the number of cited studies. 

**Author Response:**

We do not have an existing reference for this point. We have inserted one key reference to support it:

Cenciarini, M., Loughlin, P. J., Sparto, P. J., & Redfern, M. S. (2009). Stiffness and damping in postural control increase with age. *IEEE Transactions on biomedical engineering*, *57*(2), 267-275.

**Minor Comment from Editor 6:**

Lines 1005 and 1022, these sentences read odd. I guess these are supposed to be sub-headers, so in this case please format the accordingly. Otherwise, please integrate them more smoothly in the text.

**Author Response:**

We have more clearly formatted this as a sub-heading. Our sub-heading formatting in our last submission was hidden by the track changes formatting.
